# The compositional nature of number concepts: Insights from number frequencies

**DOI:** 10.1016/j.cognition.2025.106213

**Published:** 2025-06-26

**Authors:** Maxence Pajot, Mathias Sablé-Meyer, Stanislas Dehaene

**Affiliations:** a Cognitive Neuroimaging Unit, CEA, INSERM, Université Paris-Saclay, NeuroSpin center, 91191 Gif/Yvette, France; b Sainsbury Wellcome Centre for Neural Circuits and Behaviour, University College London, London, UK; c Collège de France, Université Paris Sciences Lettres (PSL), 75005 Paris, France

**Keywords:** Numerical cognition, Language-of-thought

## Abstract

The frequency with which humans use words provides a window into the psychological representation of the corresponding concepts. Capitalizing on the availability of massive lexical databases, we evaluate the frequency with which specific number words and their combinations (e.g. “twenty-four”, “quatre-vingt-douze”) are used in six different languages. We use these data to probe the hypothesis that complex concepts arise as syntactic combinations of simpler ones in a language of thought. First, we confirm our previous report of a regular and reproducible profile of decrease in frequency with number size, with local peaks for round numbers. Second, we show that frequency varies with the simplicity of the decomposition of a number into small prime factors. Third, we demonstrate that the entire frequency profile, including its overall decrease and local peaks, can be modeled by a grammar of algebraic combinations, whereby each number arises from addition and multiplication operations on smaller numbers. Those findings strengthen the hypothesis that compositionality in a language of thought underlies the emergence of exact number concepts.

## 1 Introduction

The frequency with which humans use a word can provide a window into the psychological simplicity and accessibility of the corresponding concept (Michel et al., 2011). As early as 1949, Zipf theorized that longer words were less frequent than shorter ones because of a principle of least effort: shorter words impose less cognitive load than longer ones, and are thus assigned to more frequently used concepts (Zipf, 1949). A recent review (Brysbaert, Mandera, & Keuleers, 2018) confirms that word frequencies extracted from large databases (more than 20 million words) are indeed good predictors of processing efficiency.

In the case of number words, previous work showed that across multiple languages, the frequency of number words follows a universal law (Dehaene & Mehler, 1992). Overall, log frequency decreases linearly with log number magnitude with a slope close to −2, indicating a power-law decrease of frequency with number magnitude, i.e. freq(n) $\sim \frac{1}{n^{2}}$. This overall decrease is interrupted by local peaks for round numbers such as decades. Dehaene and Mehler (1992) considered multiple possible explanations for this: sampling artifacts, mathematical regularities inherent to number notation, environmental biases, and psychological constraints on mental number representations. They established that even though the first three sources had some impact on number word frequency, they did not suffice to account for the full frequency profile. Rather, the psychological organization of number concepts, including Weber’s law, must be a driving factor in the observed frequency profile.

Jansen and Pollmann (2001) also observed local peaks in number frequencies in multiple languages, such that round numbers were more frequent than others. They attributed it to a propensity to use as approximate numbers those that were “10-round” (decades), “5-round” (multiples of five, or halves of decades), “2.5 round” (quarters of powers of ten) and “2-round” (some even numbers). Those numbers can be used in an approximation context, and therefore end up being more frequent that the others, who can only be used to refer to a precise quantity. The authors theorized that this was due to a natural capacity to manipulate numbers by multiplying them by 2 or 10, or by halving them.

As for the overall decrease in the frequencies of number words, approximated by a 1/n^2^ law, some have attributed it to a natural “need probability” of numbers (Cheyette & Piantadosi, 2020; Piantadosi, 2016): they define this “need probability” as “how often a numerosity *n* is encountered and represented”, and assume that it follows a 1/n^2^ law, such that smaller numbers are much more “needed” than larger ones in everyday life. However, this explanation does not seem fully satisfactory. First, it does not explain the specific form of the relationship, but merely assumes it.

Second, numerals in the spoken or written lexicon are cultural inventions generated by humans, and could in principle take any possible value; indeed, humans do not shy away from generating large powers such as hundred, thousand, million, billion and trillion, and even imaginary numbers and other mathematical figments of their imagination. Thus, the fact that humans generate some numbers much more frequently than others appears to us as a cultural fact that still calls for a full explanation, rather than an objective fact about the natural environment in which humans live.

In this context, the aim of this paper is threefold: to use newly available data to extend the results of previous studies on number word frequencies to any composite number word (e.g. twenty-four) rather than to single words alone; to use these data to showcase a novel finding, the importance of the decomposition of a number into small prime factors; and to propose a model that can explain the entire curve of frequencies of numbers across languages, including both the overall frequency decrease with magnitude and the occurrence of local peaks.

Our model builds on the theoretical framework of a Language-of-Thought (LoT) for number cognition introduced by Dehaene, Sablé-Meyer, and Ciccione (2025), which posits that the cognitive complexity of a number is predicted by its minimum description length (MDL), the length of the shortest expression that allows it to be constructed from smaller numbers. The Language-of-Thought, as initially proposed by Fodor, postulates that human cognition relies on a structured, symbolic mental code that enables the composition of complex representations and reasoning (Fodor, 1975). We previously used this idea in the domain of geometry, where we showed that humans remember geometric shapes more easily if they can infer a shorter mental program to represent them; we argued that simpler shapes are easier to encode and recall due to their shorter MDL, and showed that a limited language using only repetitions, concatenation, nesting as well as elementary curve tracing operations, could account for human behavioral data (Sablé-Meyer, Ellis, Tenenbaum, & Dehaene, 2022). Recently, Dehaene, Sablé-Meyer and Ciccione (2025) proposed that the same Language-of-Thought could be applied to the development of human representations of exact number. Starting from a single object, applying the primitives from the geometric LoT would generate a “syntax of sets” with intuitive concepts of addition and multiplication: concatenation of two sets of marks corresponds to addition (a set of 3 and a set of 2 make a set of 5), while nesting corresponds to multiplication (3 sets of 4 make a set of 12). Thus, an algebra of addition (including its simplest version +1, i.e. the successor function) and multiplication would generate all number concepts.

**Fig. 1:**
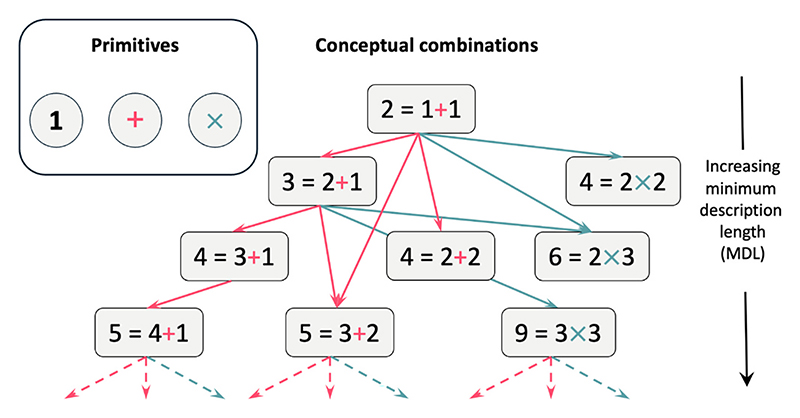
Examples of different constructions of numbers based on three primitives: 1, + and x. New number concepts can be generated by addition of +1, equivalent to the successor function (left), other additions, and multiplications (right). Multiplication provides a short-cut to larger numbers, thus explaining why they can be conceptually simpler and more frequent.

Here, we evaluate the capacity of this model to explain number frequencies. While each number can be formed from many different expressions (6 = 5 + 1, 4 + 2, 3 + 3, 3 × 2…), our model assumes that each of these representations contributes to total frequency as a function of its expression length – a combination of the costs of additions and multiplications, starting from the primitive number 1 (Fig. 1). By fitting our model to extensive frequency data, we identify the most likely expression(s) that represents each number.

## 2 Method

We used the Google n-grams database (https://books.google.com/ngrams/), which is based on Google Books, to extract the frequency of numbers in different languages. A python program using the *num2-words* library was written to automatically list number words in the range 1–99 in English, Spanish, Italian, German, Russian and French. For French, numerals over 97 were excluded since they are expressed with 4 words, which results in a 7-grams when using dashes; since the Google n-grams database is limited to 5-grams, they could not be included. The program automatically sent queries to the n-gram database. For each number, we calculated its frequency by averaging the n-grams frequencies over the years between 1950 and 2000 (very similar results were obtained from other time periods). Since composite numerals could be written with or without a dash (e.g. *twenty-three*), we queried both forms and added their frequencies. As *num2words* only provides the masculine version of the number, in languages where other feminine or neutral forms exist for some numbers (for example uno/una in Spanish), we manually queried the n-gram database to include all possible words for a given number.

We also introduced a correction for the fact that, in these raw data, some number words can appear alone as well as within other numerals. For instance, in English, decade numbers such as *twenty* appear by themselves, but also within composite numerals such as *twenty-one, twenty-two*, etc. – and conversely, unit words such as *two* can appear alone or within *twenty-two, thirty-two*, etc. By default, the n-grams database counts such productions multiple times: an instance of “twenty-two” contributes to the frequency of the bigram “twenty-two”, but also of the unigrams “twenty” and “two”. Since our goal was to estimate the frequency with which each specific *quantity* between 1 and 99 was used, we introduced a correction by subtracting from the frequency of *twenty* the instances of *twenty-one, twenty-two*, etc. and, more generally, subtracting from the frequency of any numeral the frequency of any other numerals within the range 1–99 that contained it (e.g. *quinze* vs *soixante-quinze* in French). Supplementary fig. S1 shows that this correction had a rather small impact, though, and we did not endeavor to further control for the larger context in which the words occurred (e. g. “two” in *two hundred*, or “twenty-one” in *twenty-one thousand*, etc). Not only would such a correction be negligible, but it could be argued that, in such contexts, those number words appear as multipliers of the large unit with their own specific quantity meaning, similar to *two apples or twenty-one miles* (Hurford, 1975).

Similar n-gram queries were used to obtain the frequencies of Arabic numbers 1–99. In this case, no correction was performed, as the database counts each of these as a separate token. Arabic number frequencies were obtained from English books from the same time period as the words, but virtually identical results were obtained from other languages and books.

## 3 Results

### 3.1 Fine-grained profile of number word frequencies in six languages

**Fig. 2:**
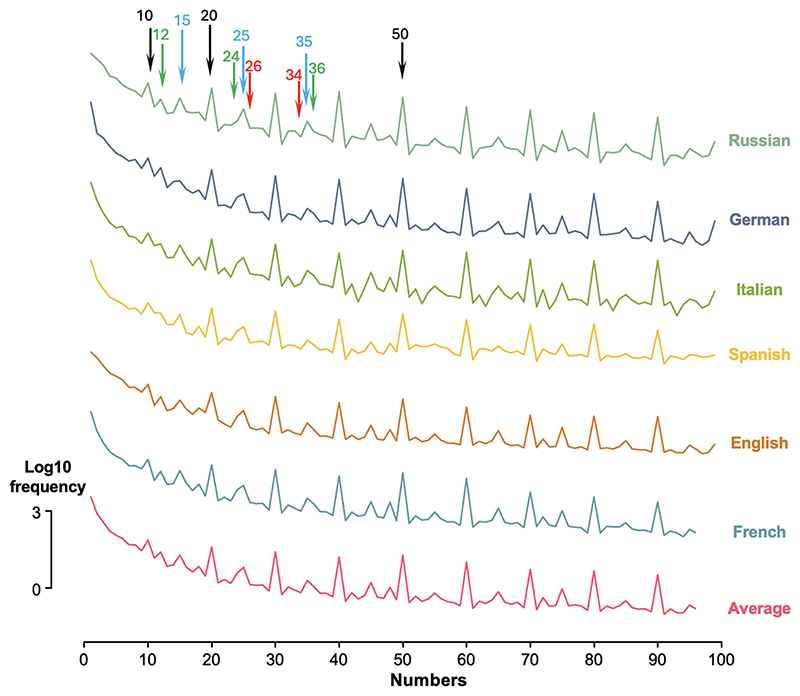
Variations in the frequency of numerals in six different languages, over the range 1–99. The frequencies are expressed in Log10 parts per million. Curves have been arbitrarily shifted for better readability. The bottom curve shows the average Log10 frequency, averaged across the six languages shown. Arrows show example multiples of 10 (black), 5 (blue), as well as examples of the contrast between numbers with small prime factors (green) and other numbers (red).

Fig. 2 shows the profile of number word frequencies over the range 1–99, on a log10 scale. The profiles show considerable similarity between each other as well as with those of Dehaene and Mehler (1992). Indeed, the minimal correlation of Log10 frequencies between any two languages is *r* = 0.97. Furthermore, a very similar profile was also obtained when counting the frequencies of Arabic numerals (Supplementary fig. S2): the correlation between Log10 Arabic numeral frequencies and the average Log10 frequencies of verbal numerals in the languages we considered was again very high (r = 0.97). The fact that our frequency profile varied little across language and notation supports the hypothesis that they reflect a universal law and approximate the frequency with which the corresponding number concepts are used, which was our goal.

Closer inspection of Fig. 2 suggests that the number frequencies exhibit a clear downward slope, replicating previous work (Dehaene & Mehler, 1992). Remarkably, all languages also seem to share the same frequency spikes, not only for multiples of 5 and 10, but also within each decade. The peaks seem to occur for numbers with simple decompositions into prime factors, such as 24 (= 2^3^ × 3) and 36 (= 2^2^ × 3^2^). In the following, we first consolidate those observations, then attempt to provide a theoretical account of them.

### 3.2 Modeling by linear regression

For simplicity, here and in all subsequent statistics, unless otherwise stated, we only report the analysis of mean Log frequency in English. However, Table 1 shows that the results were always tightly replicated in other languages.

**Fig. 3:**
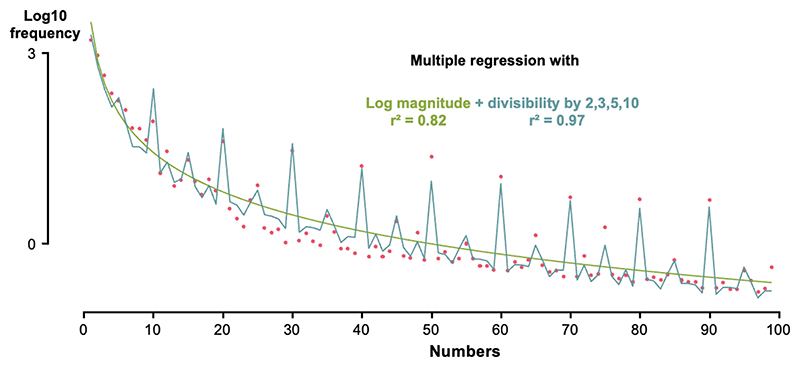
Modeling the number frequency profile by linear regression. Red dots, measured Log10 frequencies in English; Yellow curve, prediction from a linear regression with log number magnitude alone; blue curve, prediction from a linear regression with log number magnitude and 4 indicator variables for divisibility by 2, 3, 5 and 10.

As in Dehaene and Mehler (1992), a Log-Log regression showed that a very large proportion of the overall variance in Log number frequency could be accounted by the Log magnitude of the numbers (r^2^ = 0.82, *p* < 0.0001). In such a Log-Log regression, the slope was −2.053 ± 0.098 (mean ± standard error). This value implies that frequency is a power function of number *magnitude*, with an exponent around −2. However, as seen in Fig. 3, the fit was imperfect, and additional regressors were needed to account for further peaks in the frequency profile. To probe this in a systematic manner, we defined indicator variables for whether a number was divisible by 2, 3, 5, 7 and 10. We used hierarchical linear regression to examine which of these variables entered significantly in a regression, with Log magnitude as an additional factor. The results showed that there were significant effects of divisibility by 10 (β = 1.13, *p* < 10^−27^), by 5 (β = 0.40, p < 10^−9^), by 3 (β = 0.13, p <10^−3^) and by 2 (β = 0.12, p < 10^−3^), in this order. Divisibility by 7 was not significant (β = −0.01, *p* > 0.9). Those regression results were very consistent across languages (Table 1). While the most prominent local peaks were for multiples of 5 and 12, a regression without those numbers still yielded 2 and 3 as significant predictors (*p* < 0.05), suggesting that this analysis is not purely driven by the high frequencies of multiples of 5 and 12.

**Fig. 4:**
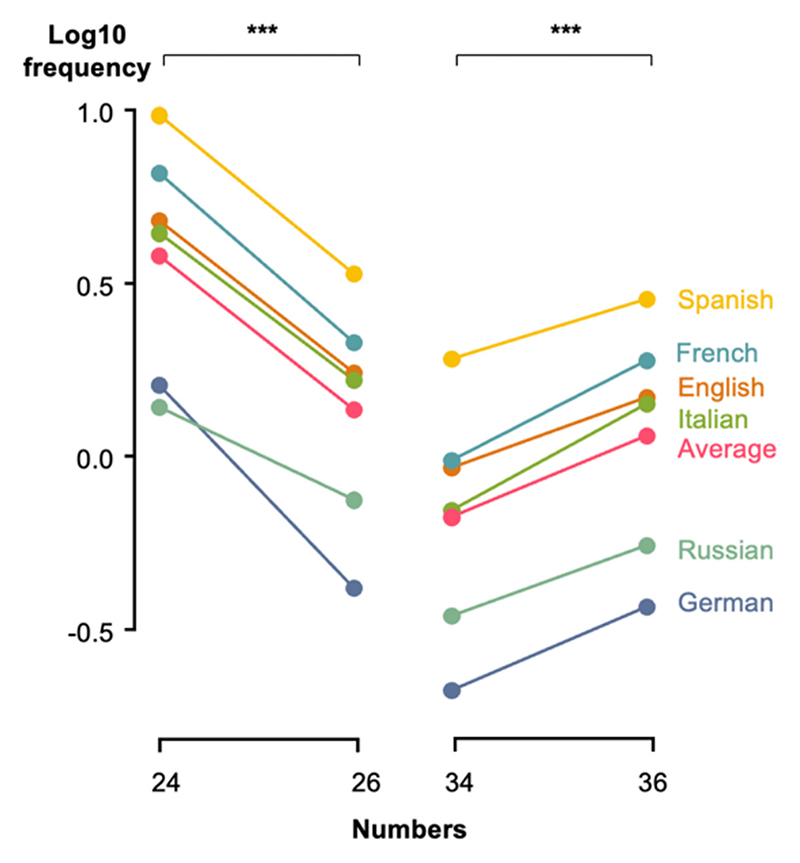
Example of variations in number frequency independently of number size and individual number words. The numbers 24 and 36, which have a lot of small divisors, are more frequent that the corresponding numbers 26 and 34 (in six different languages). Note that those numbers use the same individual number words overall (four, six, twenty, and thirty).

Our theory predicts that frequency relates to the simplicity of a number’s decomposition into smaller numbers. Thus, numbers with comparable magnitudes but more complex decompositions into prime factors should have significantly lower frequencies – even if they use the very same component words. As an example, we observe that, in all languages, 24 (= 2^3^ × 3) is more frequent than 26 (= 2 × 13), and conversely 36 (= 2^2^ × 3^2^) is more frequent than 34 (=2 × 17) (Fig. 4). To test this, we compared the log frequencies of the pairs 24 versus 26, as well as 34 versus 36. A paired *t*-test across languages showed that, in both cases, the difference was highly significant in the predicted direction (*p* < 10^−4^). Since the very same words *four, six, twenty* and *thirty* are used to compose these numerals, those differences cannot be attributed to differences in the representation of individual words, but must arise from a genuine influence of their numerical values. Similarly, the same effect holds true for 84 vs 86 and 94 vs 96: 84 (= 2^2^ × 3 × 7) is more frequent than 86 (= 2 × 43) and 96 (= 2^5^ × 3) is more frequent than 94 (= 2 × 47) (both *p* < 0.05).

We next examined whether a better fit could be achieved by considering the multiplicities of prime factors, and not just their presence, in the decomposition of a given number. In the overall regression, this was not the case. Model comparison showed that the model with multiplicities was not better than the model with divisibility alone (ΔAIC > −10). We also redid this analysis while limiting the analysis to the more homogeneous case of all two-word numbers between 21 and 99. Results did not vary significantly: there was a small improvement of using the multiplicity instead of the divisibility for English (ΔAIC = −19), but not for the other languages (ΔAIC > −10). Table S1 provides details of the differences between the use of multiplicity and divisibility. Even though there seemed to be a small improvement when using multiplicity rather than divisibility, the lack of consistency across all languages makes it hard to conclude.

Could some of these phenomena arise from surface properties of the number words, such as their length, rather than from deep arithmetic properties of the numbers themselves? According to Zipf’s law, shorter words are more frequent than longer ones, and we indeed find that decades (which are expressed with a single word) are markedly more frequent than other numbers of similar magnitude (which are expressed with two words or more). The case of 24 vs 26 and 34 vs 36, however, suggests that this explanation may not suffice. Indeed, we found that the above multiple regressions were barely changed when adding a regressor for total word length (number of characters, including dashes). In this regression, Log magnitude and all divisibility variables remained significant.

### 3.3 Modeling the combinatorial origins of number concepts

The significant linear regression of number frequency with Log magnitude and divisibility, in six different languages, suggests that the factorial composition of a number plays a role in its frequency. However, distinct regressors were separately needed to model the effects of magnitude and of divisibility. In the following, we introduce a theoretical framework that can account for the full frequency profile in one sweep, including the overall decrease in frequency with magnitude as well as the local peaks for numbers that have a simple decomposition into small prime factors.

Capitalizing on the language of thought (LoT) previously introduced for geometric shapes (Sablé-Meyer et al., 2022), Dehaene et al. (2025) suggested that numbers could be constructed by starting with the number 1 and forming sets by concatenation (a set of *n* and a set of *m*) and by recursion (*n* sets of *m*). The former implies addition (*n* + *m*), and the latter multiplication (*n* × *m*). According to this LoT, every integer can be constructed from number 1 using successive additions and multiplications. For example, 2 is built as 1 + 1, and 3 as 2 + 1 = (1 + 1) + 1. The minimum description length is then the cost of the simplest expression that yields a given number (Fig. 1). Usually, cost is just the number of symbols used, but here we assume that each symbol (1, +, x) can have a distinct cost. This hypothesis is identical to the hypothesis that, in a probabilistic context free grammar (PCFG), each production rule (here, *p* = *1, p* = *n* + *m* and *p* = *n* × *m*) can have a different probability.

**Fig. 5:**
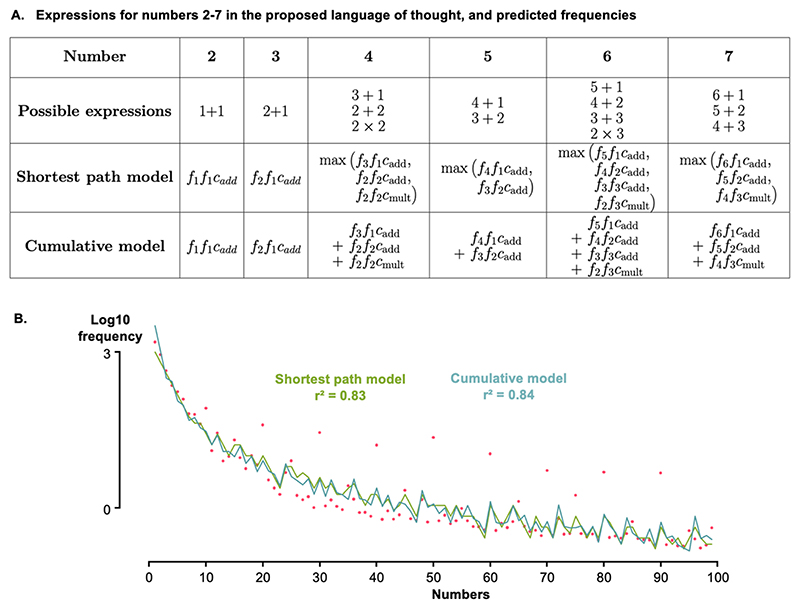
Modeling number frequencies from first principles, assuming that number concepts arise from algebraic combinations in a language of thought. **A**. Each number can be expressed using multiple algebraic expressions that combine smaller numbers using addition and multiplication. Two models are proposed: one in which only a single expression, with the shortest path to smaller numbers, and therefore maximal predicted frequency, determines the frequency of the new number (shortest path model); and another where that frequency is predicted as the sum over all possible expressions (cumulative model). **B**. Plots of observed data (red dots) and model predictions for the shortest path model (green curve) and the cumulative model (blue curve). The whole curve, except for the multiples of 10, is accurately modeled.

We further assume that the frequency of a number p is the product of the frequencies of the two numbers *n* and *m* and the cost of the operator used to combine them (+ or ×) (again, this hypothesis is identical to how probabilities compose in a PCFG). For instance, since 2 = 1 + 1, the frequency of 2 is computed as the frequency of 1 multiplied by the cost of the addition operation and by the frequency of 1 again. Similarly, the frequency of any larger number depends on the frequencies of its components and the costs of the operations that combine them. See Fig. 5A for the decompositions of the first few numbers, and their corresponding predicted frequencies.

If only the successor operation +1 was used, then each number *n* would be expressed as 1 + 1 + 1 … + 1 (*n* times). Thus, the frequency of a number *n* would be given by the frequency of 1 power n, times the cost of addition power n-1. As a result, a LoT model based solely on addition predicts that log frequency should decrease linearly with n, with the slope that depends only on the frequency of 1 and the cost of addition. Such a decrease would be much faster than the −*2 Log(n)* trend which is empirically observed. In our algebraic LoT model, what enhances the frequency of larger numbers is the presence of multiplication. For all numbers greater than 3, the multiplication operation yields multiple ways to define a single number (for example 4 = 2 + 2 = 3 + 1 = 2 × 2…), some of which offer a much shorter path to 1 than a mere concatenation of +1 operations. As a result, the frequency of numbers with simple decompositions into prime factors is elevated, and in turn, through addition, the frequency of their successors is also increased.

Specifically, we considered two models. In the first, called “shortest path model”, the frequency of a number results from whichever expression has the lowest cost, and thus the highest predicted frequency. In practice, for a given number *n*, we examine each pair of integers (*i*,*j*) such that *i* + *j* = *n*, and keep the pair for which the product of the frequences and the cost of the addition, f_i_f_j_c_add_, is maximal. Similarly, we examine each pair of integers (*i*,*j*) such that *i x j* = *n*, and keep the pair such as f_i_f_j_c_mul_ is maximal. The largest of those two values is the predicted frequency for *n*:

$$f_{n}=\text{max}\left[\underset{i+j=n}{\text{max}}\left(f_{i}f_{j}c_{\text{add}}\right),\;\underset{i\times j=n}{\text{max}}\left(f_{i}f_{j}c_{\text{mul}}\right)\right]$$

The model has only three free parameters: f_1_, cost_add_ and cost_mult_, from which the frequencies of all other numbers can be computed.

To compare the model with the data, we first considered a naïve, parameter-free approach whereby the parameter values are fixed by the first few numbers, for instance f_1_ = freq(1), cost_add_ = freq(2)/freq(1)^2^ and cost_mult_ = freq(6)/(freq(3) × freq(2)), and then all of the other frequencies are predicted. This approach is already sufficient to account for the overall power law of number frequencies (Log-Log regression of frequency with magnitude, r^2^ = 0.49, slope = −1.83; see Fig. S3 for a graphic representation of the fit). However, this approach tends to overestimate the frequency of large non-decade numbers, perhaps because it implicitly relies on the assumption that multiplication wins over addition already starting at number 6 (=3 × 2). There are other reasonable choices such as cost_mult_ = freq(8)/(freq(4) × freq(2)) and cost_mult_ = freq(9)/freq(3)^2^, and as seen in Fig. S3, they do reduce the gap with actual frequencies, and better capture the observed 1/n^2^ law (respectively r^2^ = 0.54, slope = −1.85; and r^2^ = 0.84, slope = −2.13).

Although this naïve approach yielded decent results for English, it is overly sensitive to the frequency of “one” and thus did not work well for languages other than English where the word for “one” (un, uno) is the default determiner in addition to being a numeral. To alleviate this problem, we optimized the three free parameters by minimizing the mean squared error between the predicted log-frequencies and the actual log-frequencies over the full range of numbers 1–99. We used the Nelder-Mead method in the *scipy* library (Virtanen et al., 2020), using as initial point the values of f_1_, cost_add_ and cost_mult_ arising from the naïve model. Fig. 4B shows the fit of the resulting model (r^2^ = 0.83). Most of the variance in the data was captured, including the log magnitude decrease and many local peaks. Using the same linear regression on the predicted frequencies as we did on the real data, we observed significant effects of log magnitude, divisibility by 2, 3 and 5, but not divisibility by 10 (see Table 1).

### 3.4 The importance of approximation

While promising, this result sheds light on a key weakness of our modeling approach, clearly visible in Fig. 5B: the model fails to predict the spikes in frequency at multiples of 10 and, to a lesser extent 5. Indeed, our model treats 5 and 10 as any other number, constructed from recursive operations on 1, and therefore more complex than, say, numbers 4 or 8. This failure is perhaps not unexpected, because many authors such as (Chrisomalis, 2010) attribute the unique prominence of the numbers 5 and 10 in numeration systems worldwide to a non-mathematical factor, the anatomy of the human hand (five fingers). Our LoT model is blind to this property of number 5. To solve this problem, a possible method would be to use 5 and 10 as intermediate primitives, as was done in previous work on a LoT to represent exact number concepts (Denić & Szymanik, 2022). We explored variants with two additional free parameters, f_5_ and f_10_, with the idea that our LoT model would then propagate the larger frequencies for 5 and 10 to their multiples. Even though this approach improved the fit for multiples of 5, it did not do so for multiples of 10. This was because, when the frequencies of multiples of 10 are high, the model also predicts elevated frequencies for nearby numbers of the form 10n + 1, 10n + 2, etc. Yet these numbers are nowhere as frequent as their corresponding decade – indeed they are often an order of magnitude lower in frequency. Consequently, when optimizing for an additional parameter f_10_, the model privileged a lower frequency of 10 and again failed to capture the high frequencies of decade numerals.

This observation suggests that the elevated frequency of decade numbers, universally seen in all languages, arises from a distinct source – otherwise, according to our LoT, the frequencies of decades+1 would also have to be elevated. We realized that our analysis relied on a strong assumption: the frequency of each number serves as a proxy for the simplicity of the mental representation of that *exact* number. This hypothesis, however, overlooks the fact that another phenomenon occurs: decade numerals are often used to express *approximate* quantities. To further validate this fact, we examined the frequencies of the phrases “about *n*” versus “exactly *n*” for values of n between 1 and 100. Following normalization, we performed a paired *t*-test between the frequencies of some numbers in those two contexts. For numbers of the form 10*n* and 10*n* + 5, the Log10 frequencies of the “about *n*” phrase were significantly higher than those of the “exactly *n*” phrase (*p* < 0.01; Log10 difference = 0.16, corresponding to a 1.5 times larger frequency). Conversely, numbers of the form 10n + 1 and 10n – 1 were more likely to occur with “exactly” than with “about” (*p* < 10^−4^ for 10*n* + 1, *p* < 0.05 for 10*n* - 1).

The use of approximation suggests that the frequency of certain numbers (mostly decades) is elevated because the corresponding numerals are often used in an approximation context. We also reasoned that the frequency of other nearby numbers, conversely, might have been underestimated, because instead of being precisely named, they were occasionally approximated by the nearest decade. In support of this possibility, we observed that, in all languages, numbers of the form 10*n* + 1 have lower frequencies that those of the form 10*n* + 2 (paired t-test across languages and for n between 1 and 9, d.f. = 53, p < 10^−20^) and, most crucially, in all languages except Italian, lower frequencies than numbers of the form 10*n* + 3, which have the same parity (*p* < 0.005). Since numbers of the form 10*n* + 1 have a similar or even shorter representation in the LoT, this finding cannot be explained by the LoT alone. We propose that the frequency of those numbers is lower because when those quantities appear, they are more likely to be rounded to the nearest multiple of 10.

How could this approximation effect be formally modeled? In agreement with the theory of rational speech acts (Frank & Goodman, 2012), we assumed that locutors experience a trade-off between numerical precision and utterance length. On a fraction of trials (which is an additional free parameter in our model), instead of producing a precise numeral such as “twenty-three”, they opt for a shorter numeral such as “twenty” which is almost as good at communicating to the listener the appropriate quantity. To formalize this notion of goodness-of-approximation, we adopted the assumptions of previous models of the approximate number system (Dehaene, 2007; Nieder & Dehaene, 2009) and assumed that the probability of approximating is a Gaussian function of the distance between the two quantities (here 23 versus 20) on a log scale.

$$P_{\text{approx}}\left(n,n^{\prime }\right)=\kappa \cdot \text{exp}\left(-\frac{1}{2}{\left(\frac{\text{log}(n)-\text{log}\left(n^{\prime }\right)}{w}\right)}^{2}\right)$$

In this equation, κ is a free parameter modulating the overall probability of using approximation. To avoid adding yet another free parameter to the model, we took the Weber fraction *w* to be w = 0.15, as estimated from previous studies of approximate numerosity perception in educated adults (Piazza, Pica, Izard, Spelke, & Dehaene, 2013).

Formally, therefore, starting from the frequencies predicted by the LoT model alone (denoted f_n_), the approximation model generates new predicted frequencies f_n_’ through the following algorithm:

$$\begin{array}{l} \;\text{for}\;\text{each}\;\text{decade}\;\text{number}\;n\;\text{do}\;\\ |\begin{array}{l} \;\text{for}\;\text{each}\;\text{initial}\;\text{number}\;p\neq n\;\text{do}\\ \;|\;f_{p}^{\prime }=f_{p}\times \left(1-P_{approx\;}(n,p)\right)\\ \;f_{n}^{\prime }=f_{n}+\sum_{p}\left(f_{p}-f_{p}^{\prime }\right) \end{array} \end{array}$$

**Fig. 6:**
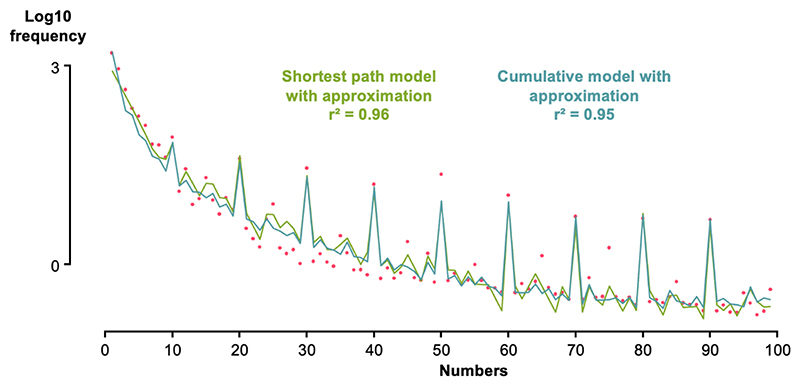
Modeling number frequencies from first principles, assuming that shorter, numerically close number words can substitute for the exact number word. Plots of observed data (red dots) and model predictions for the shortest path model (green curve) as well as the cumulative model (blue curve). Introducing an additional optimizable parameter -the probability of using an approximation- significantly improves the fit in both cases. This is particularly evident for multiples of 10, whose frequencies could not be adequately explained by the LoT model alone.

We used this approximation model to match the English frequency data. All four free parameters, including the additional parameter κ, were simultaneously fit. The resulting curve, shown in Fig. 6, reached an excellent fit for both decade and non-decade numbers (r^2^ = 0.96). Furthermore, a linear regression on the predicted frequencies, using the same predictors as above (Log magnitude and divisibility by 2, 3, 5 and 10) attained a r^2^ of 0.98, with all predictors being significant (*p* < 0.005), as in the actual data (see Table 1). This includes divisibility by 10, which was not significant in the previous model.

To ensure that these good fits did not arise solely from the flexibility of our model, which relied on four continuous parameters to minimize the MSE loss, we used the same procedure to fit identical models to shuffled number word probabilities. We fitted 1000 such models, all but one of which performed worse than our model on real data. From this, we derive a non-parametric *p*-value of *p* = 0.001 for the overall fit. The quality of the fit was not due to some peculiarities of the English language, as the same model resulted in equally good fits for other languages as well (Fig. S4).

### 3.5 Cumulative variant of the model

In the previous model, we assumed that each number inherits its frequency from a single expression in the LoT. However, another plausible option is that the same number concept can be represented by multiple mental expressions. It seems likely that, the higher the education level, the more diverse and flexible is the repertoire of stored facts for the same number (Dehaene, 2011; Dehaene et al., 2025). For instance, educated adults know that 10 is 2 × 5, but also 6 + 4, 7 + 3, etc. (complements to 10). Depending on the problem at hand, one representation may be more useful than the others (e.g. to solve 6 + 7, it may be useful to retrieve 6 + 4 = 10 and 7 = 4 + 3). Thus, each number is likely to be associated with a flexible repertoire of tightly integrated mental representations.

We therefore considered here a cumulative model whereby the frequency of a number *n* is the sum of the frequencies of all the possible combinations that can make the number *n*. As before, because 2 = 1 + 1, the frequency of 2 was computed as the frequency of 1 squared, multiplied by the cost of the addition operation. However, starting at number 4, the frequency count began to change, as 4 can be written 3 + 1, 2 + 2, or 2 × 2. In this variant of the model, we summed these contributions, each weighted by their complexity. As explained in Fig. 5A, the frequency of a given number n was then given by:

$$f_{n}=\underset{i+j=n}{\sum }\left(f_{i}f_{j}c_{\text{add}}\right)+\underset{i\times j=n}{\sum }\left(f_{i}f_{j}c_{\text{mul}}\right)$$

To predict the whole curve, we again started from the 3 parameters (f_1_, cost_add_, cost_mult_), and iteratively generated the predicted frequencies for the first 100 numbers. The parameters were optimized to minimize the mean squared error (MSE) between the predicted and observed log-frequencies.

As shown in Fig. 5B, this cumulative model alone yielded a good fit, with a resulting r^2^ of 0.84. As in the previous shortest-path model, a linear regression on predicted frequencies revealed significant effects of log magnitude (with a slope of −2.10) and of divisibility by 2, 3, and 5, while divisibility by 10 remained non-significant. Thus, cumulative and shortest-path models were on a par in predicting observed frequencies, and none suffice to explain the higher frequency of decades.

As with the shortest-path model, and using the same equations, we added an optimizable parameter *κ* to account for the use of decade number words in an approximation context. This cumulative model with approximation achieved a r^2^ of 0.96, accurately fitting the whole curve, including multiples of 10 (see Fig. 6). Contrary to the shortest path model, a linear regression on the predicted frequencies, using the same predictors as above (Log magnitude and divisibility by 2, 3, 5 and 10), was not significant for divisibility by 5. Still, all other predictors were significant (*p* < 0.001), and the regression attained a r^2^ of 0.98.

**Fig. 7:**
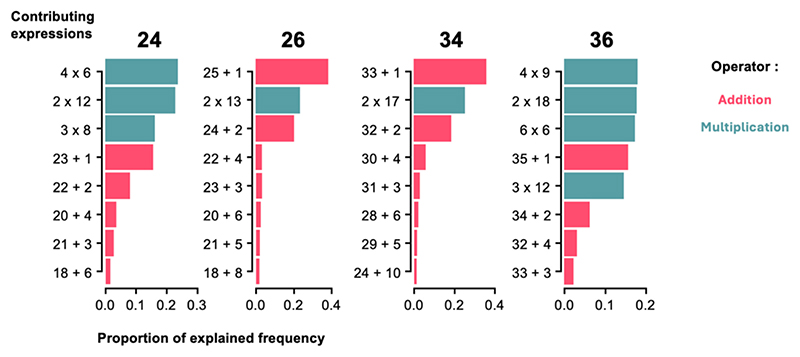
Contributions of different operations to the predicted frequencies of numbers 24, 26, 34, 36. For each number, the graph shows the eight expressions that, according to the cumulative model, contribute the most to its frequency. Expressions are ordered by the proportion of the total frequency that they explain (within the sum of Fig. 5A). 26 and 34 are best explained by the successor function +1 (top line), but 24 and 36, which have multiple small divisors, are best explained by multiplicative expressions (blue).

Additionally, the cumulative model allowed us to delve into the contributions of each operation to the predicted frequencies: once the parameters of the model were fit, we could sort the different expressions for each number according to their predicted frequency. This is illustrated in Fig. 7 for the numbers 24, 26, 34 and 36, with the parameters of the cumulative model with approximation. Bars represent the eight expressions that contribute most to total predicted frequency, ordered by their own frequency. For numbers 26 and 34, the simplest representation is based on the successor function, i.e. these numbers are expressed primarily as the preceding number plus one. However, for numbers 24 and 36, which have a simple decomposition into small prime factors, expressions involving multiplication, such as 24 = 4 × 6, systematically come on top: the low cost of multiplication provides a “shortcut” to those large numbers. This observation explains our finding that 24 and 36 have higher frequencies than 26 and 34 (Fig. 3). While the primary representation of 26 and 34 is through the successor function, 24 and 36 benefit from additional representations that make use of their many divisors.

## 4 Discussion

Using extensive data from Google n-grams, we successfully reproduced and extended previous results on the frequency of number words (Dehaene & Mehler, 1992; Jansen & Pollmann, 2001; Piantadosi, 2016). A log-log regression, now including all 1- and 2-digit numbers between 1 and 99, confirmed that number magnitude was a major predictor of number frequency. The log-log slope of approximately −2 confirmed that number frequency follows a power law and decreases approximately as 1/n^2^. We also replicated and extended the local peaks in frequencies for round numbers reported in (Dehaene & Mehler, 1992; Jansen & Pollmann, 2001).

While previous work on this topic had to rely on painstakingly crafted single-word frequency tables based on a limited corpus, the present work leverages a massive dataset with more than 500 billion words, the google books corpus (Michel et al., 2011). This explains why the results were very robust, across multiple languages and different time periods. Google’s n-gram database proved to be a valuable tool, and further works could use it even more thoroughly, for instance by searching for the frequencies of number words in certain contexts.

This high-quality data allowed us to discover a new phenomenon: the role of a number’s decomposition into prime factors in its frequency. In linear regression, in addition to log number magnitude, the presence of integer divisors 2, 3, 5 and 10 led to a large and significant increase in frequency. In these regressions, we also compared the predictive power of mere divisibility by a number, compared to the multiplicity of that number. Although there seemed to be some improvement in using the multiplicity, especially when looking only at two-words numbers, this was not consistent across all languages, making it hard to argue either way.

All of these observations could be explained by the Language-of-Thought hypothesis for exact number concepts recently put forward by Dehaene et al., 2025. We proposed a simple quantitative model for number frequency, which assumes only that each number can be formed through an addition or multiplication of two smaller numbers, and that the length of its expression predicts frequency. Two variants of this model were proposed: one where number frequency arises solely from the shortest possible expression, the other where the frequency of each number arises from the sum of the frequencies of all possible operations that yield it. Both approaches yielded similarly good results: they captured not only the 1/n^2^ decrease in frequency, but also the local peaks observed for multiples of 2, 3 or 5. Based on the quality of their fit to our data, neither model seems superior to the other. However, the two models offer distinct perspectives on how exact number concepts are formed. The shortest path model, which posits that the preferred expression is the shortest one, is most closely aligned with the broad literature on the role of MDL or, equivalently, the “simplicity principle”, in various aspects of human cognition (Chater & Vitányi, 2003; Feldman, 2003). The cumulative model, on the other hand, relies on the very likely assumption that each number can have several mental representations, arising from the many different expressions that yield that number. Indeed, it seems almost certain that most educated humans possess multiple representations of some numbers: at a minimum, all know the successors of numbers (e.g. that 8 comes after 7) and some basic divisibility properties (e.g. that 8 is 2 times 4). In contexts involving fast calculation, having access to multiple equivalent expressions can be advantageous. For instance, remembering complements to 10 helps solve subtractions such as 14–6. We thus speculate that mastery of a larger repertoire of representations for the same number may be a good predictor of arithmetic efficiency (Dehaene et al., 2025), a prediction that remains to be quantitatively evaluated.

The only deficiency of our model, common to both variants, was that it did not accurately predict the elevated frequency of decade numbers (multiples of 10). The data indicates that, in natural language use, these numbers are used at least 10 times more frequently than their neighbors. Since this frequency boost does not extend to nearby numbers such as their successor, it cannot easily be explained by our approach, where the frequency of *n* + *1* cannot be far from that of *n*. Instead, we propose that the distinct frequency profile of decade numbers is due to their specific use in an approximation context (Jansen & Pollmann, 2001). This is in line with much literature on number cognition and the approximate number system: in all sorts of contexts, whenever participants are asked to name a number of which are they are uncertain, they approximate it by resorting to a multiple of the base (or of one of its powers) which is close enough to the distribution of potential answers (e.g. Ciccione, Sablé-Meyer, & Dehaene, 2022; Izard & Dehaene, 2008). This behavior makes sense in our theory: given their uncertainty, people provide a number close enough to the meaning they intend to express, yet with a short verbal expression – and in the range 10–99, decade numbers have the shortest word length.

In other words, the number frequency data suggests that two distinct factors are at work: the complexity of the internal expression of the number as a concept (which, according to us, is given by its expression using the primitives 1, + and ×), and the length of its external expression as a numeral (its word length). In an exact number context, the first factor dominates; but in an approximation concept, participants resort to approximation behavior. In accordance with the theory of the rational speech act (Frank & Goodman, 2012), to express a given quantity, they opt for the shortest utterance length, while still conveying a close-enough numerosity. Following these ideas, we found that the fit of the model could be vastly improved by adding another parameter: the probability of approximating a number by a multiple of 10. This probability was modulated by a gaussian of the distance between these numbers on a log-scale, following previous work on another well-documented part of human number cognition: the approximate number system (Dehaene, 2007, 2011; Piazza et al., 2013). Once these factors were included in the model, the full frequency curve, including peaks at decades, was faithfully captured (Figs. 6 and S4).

An added benefit of our LoT model is that it provides a precise hypothesis about the mental representation of each number. To find the representation of a number in the LoT, a naïve approach would be to take the representation with a minimal number of operations needed to reach that number, starting from the primitives 1, + and ×. This would correspond to the simplest approach in the LoT literature, which considers only the most compact expression for a concept, the one with the shortest MDL (Dehaene, Roumi, Lakretz, Planton, & Sablé-Meyer, 2022). However, applying this approach here would have the major drawback of supposing that adding and multiplying two numbers bring about the same psychological complexity. Here, we sidestepped this issue by letting these costs be variables (f_1_, cost_add_ and cost_mult_) that could be fit to the entire data set.

Could our observations be explained by alternative non-psychological factors? We cannot exclude the possibility that the higher frequency of round numbers and multiples of the numbers 2, 3 and 5 arises, at least in part, from their nicer mathematical properties. For instance, some of these numbers allow for an easier geometric disposition in a rectangular box (this is why eggs come in baskets of 6, 12, 24 or 36, never 7 or 13). Numbers with a simpler decomposition into prime factors could have been selected because they facilitate division and sharing – this is likely to be the reason for the choice of base 60 in ancient Babylon, which continues to influence our choice of 60 minutes for the hour. However, Dehaene and Mehler (1992) already pointed to the fact that large numbers get produced because of the simplicity of their mental representation, even when these numbers are wrong. For instance, a “centipede” [hundred legs] is a “mille-patte” [thousand legs] in French, yet some species have as few as 28 legs! Similarly, when it comes to geometry, humans do not always seem to select the best configurations. For instance, triangular boxes with 3, 6, 10 or 15 eggs, or hexagonal boxes with 7 or 19 eggs would yield a more compact spatial arrangement than our usual rectangular boxes; the fact that such geometric shapes seem less felicitous is a psychological fact that can, in turn, be explained by a Language-of-Thought for geometry (Dehaene et al., 2022; Sablé-Meyer et al., 2022). More generally, the present research, in contrast with the Platonic conception that views mathematics as a reality independent of the human mind, squarely affirms the role of psychological and cultural factors in the construction of numbers. Consequently, the reverse inference should be possible: analyzing numbers and their properties provides a window into the mind that created them (Carey, 2009; Chrisomalis, 2010; Dehaene, 2011; Lakoff & Núnez, 2000). A great advantage of our LoT approach is that it provides a single, unified account of these frequency peaks as well as the overall decrease of log Frequency with log magnitude, which would otherwise have to be imputed to a distinct, underspecified and yet universal “need” function that privileges small numbers (Piantadosi, 2016).

Similarly, parts of the curve could be explained by chunking into chunks of 5, 10 or 12, as most local peaks occur at multiples of these numbers. However, such chunking would not explain the observation of elevated frequencies for other numbers, such as powers of 2. More crucially, chunking would not explain the entire frequency curve, as we do here, but merely the presence of peaks that deviate from the 1/n^2^ curve. Furthermore, while chunking by 5 and 10 can be naturally linked to our ten fingers, the recurrence of 12 across languages would also need to be explained, whether through the concept of a dozen or the division of the day into 12-hour periods. The omnipresence of 12 in all languages, whether it is with the use of the word dozen, or the fact that it is the length of half a day in hours, is itself a cultural phenomenon that needs an explanation. This phenomenon is explained by the Language-of-Thought, but is merely postulated by the chunking model.

Our theory builds upon a long tradition of research in number cognition and the Language-of-Thought, and can be seen as an integration and prolongation of several previous proposals. First, a particularly influential theory of exact number acquisition emphasizes the critical role of the successor function (Carey, 2009, 2011; Piantadosi, Tenenbaum, & Goodman, 2012). This operation, which enables the generation of each number as an increment (+1) from the previous one, is universally thought to provide a foundational cognitive operation for the concept of exact number. The operation is seamlessly incorporated into our LoT framework, where +1 emerges as the least computationally demanding operation among the available primitives. Indeed, in our cumulative model, we found that the successor function plays a crucial role in predicting frequencies. For many numbers, it is the operation with the single greatest contribution to overall frequency, with exceptions occurring only for numbers with simple prime factor decompositions.

Second, our theory builds upon a previous proposal by Piantadosi, Tenenbaum and Goodman (Piantadosi, 2023; Piantadosi et al., 2012), who introduced a formal model for the acquisition of counting based on a LoT similar to a programming language, with primitives of arithmetic, lambda calculus and logic. The model provided a cogent account of how children go from subset knowers to cardinal principality knowers (Piantadosi et al., 2012). Their proposal, while related to ours, differs in several ways. First, it only models a specific task (how children match number words to sets in the “give a number” task), not the emergence of abstract concepts. Another difference is that the language has primitives of *singleton, doubleton* and *tripleton*, essentially assuming exact concepts for numbers 1, 2 and 3, whereas in our model, these are composite concepts formed using only the set with one element and concatenation. Third, conversely, an important primitive in our LoT that is absent in Piantadosi’s is the concept of *set of sets* (i.e. a recursive concept of set) that naturally accounts for the role of multiplication in the origins of larger number concepts. However, these differences could easily be reconciliated, and the two approaches are quite similar in spirit.

This present proposal is also deeply related to other theories for the origins of exact number cognition. As further detailed in Dehaene, Sablé-Meyer, and Ciccione (2024), it can be seen as a formalization and implementation of a previous proposal by Guerrero and Park (2023), who also stress the importance of composing number concepts from limited primitives using nested operations of addition and multiplication (see also Guerrero, Hwang, Boutin, Roeper, & Park, 2020). Spelke (2017) also proposed that number concepts originate from syntactic combinations that amount to these two operations, but using sentences in natural language instead of an internal Language-of-Thought.

Relative to these previous proposals, our model has the advantage of simplicity (only 3 primitives), precision, and quantitative fit to actual data. By identifying the three or four optimal parameters that best fitted the number frequency profile, we obtained a simple and precise proposal for the mental representations of each exact number. For instance, we found that 24 is likely to be represented as 4 × 6, but also as 2 × 12 or 3 × 8 or as the successor of 23, in this order (see Fig. 7 for other examples). It is remarkable that such a precise hypothesis could be obtained solely from number frequency data. In the future, it will be important to test it empirically, for instance using behavioral measures of number priming (e.g. Galfano, Rusconi, & Umilta, 2003; Jackson & Coney, 2005; LeFevre, Bisanz, & Mrkonjic, 1988).

## Supplementary Material

**Appendix A.Supplementary data**

Supplementary data to this article can be found online at https://doi.org/10.1016/j.cognition.2025.106213.

Supplementary material

## Figures and Tables

**Table 1:** Result of multiple regressions on Log10 frequency with Log10 number magnitude and 5 indicator variables for divisibility by 10, 5, 3, 2 and 7. In all languages, all regressors were significant, with very similar regression weights across languages, except for divisibility by 7 which was introduced as a control. The four bottom lines show the same analysis applied to the predictions of the best-fitting shortest-path and average models of English frequencies, with and without approximation.

Language	r^2^	Log10 magnitude		Mult10		Mult5		Mult3		Mult2		Mult7	
		β	*P*-value	β	P-value	β	P-value	β	P-value	β	P-value	β	P-value
**English**	0.974	−2.09	9.09E-72	0.65	6.66E-13	0.47	1.52E-13	0.13	1.69E-04	0.12	8.41E-04	−0.00	0.927
**French**	0.977	−2.32	6.28E-72	0.72	8.40E-14	0.49	1.91E-13	0.12	0.001	0.16	4.56E-05	−0.01	0.818
**Italian**	0.966	−2.36	2.19E-66	0.69	7.92E-10	0.55	7.08E-12	0.15	7.21E-04	0.24	4.12E-07	0.02	0.753
**Deutsch**	0.964	−2.59	6.40E-66	0.94	7.67E-13	0.42	4.55E-07	0.13	0.008	0.11	0.029	0.03	0.645
**Spanish**	0.941	−1.74	3.36E-55	0.71	2.54E-10	0.33	6.48E-06	0.10	0.021	0.11	0.014	−0.02	0.713
**Russian**	0.963	−2.20	4.03E-64	0.82	1.58E-12	0.58	5.60E-13	0.12	0.006	0.15	0.001	0.01	0.864
**Average**	0.978	−2.23	1.52E-72	0.74	2.06E-15	0.49	1.71E-14	0.11	9.98E-04	0.16	8.97E-06	0.01	0.875
**Shortest path model**	0.982	−2.08	9.27E-82	- 0.09	0.144	0.19	1.52E-05	0.14	2.16E-07	0.1	3.87E-04	0.01	0.753
**With approximation**	0.976	−2.11	1.43E-74	0.8	2.70E-18	0.18	5.79E-04	0.14	1.09E-05	0.09	0.004	0.03	0.477
**Cumulative model**	0.986	−2.1	2.21E-86	0.01	0.783	0.07	0.068	0.12	1.16E-06	0.2	3.09E-13	0.04	0.217
**With approximation**	0.986	−2.02	2.71E-85	0.95	2.00E-31	0.05	0.226	0.07	0.002	0.11	8.42E-06	0.05	0.139

## Data Availability

We used publicly available data. The code is available on GitHub: https://github.com/MaxencePajot/number_frequencies.
