# Supplementary material for: The compositional nature of number concepts: Insights from number frequencies
Source: Cognition. Author manuscript; Available in PMC 2025 Nov 24. (PMC7618399; doi:10.1016/j.cognition.2025.106213)
Supplement: Supplementary material [file EMS210440-supplement-Supplementary_material.docx]

Supplementary material


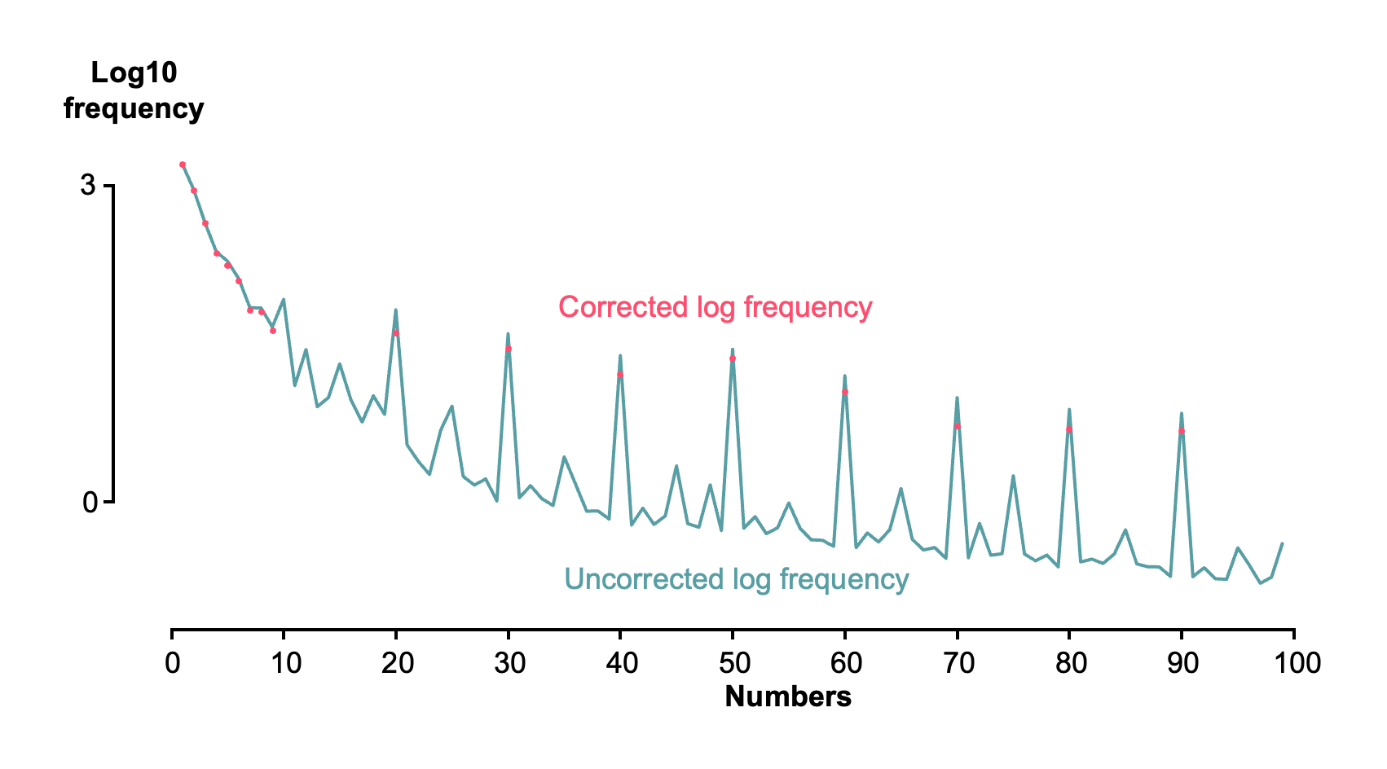


**Figure S1.** Negligible size of the variations in the observed frequencies of numerals before and after correcting for words that appear in multiple numerals (for instance after subtracting the frequency of “twenty-one”, “twenty-two”, etc., to the frequency of “twenty”). This figure shows the frequencies of English numerals, but all languages were corrected in the same spirit.


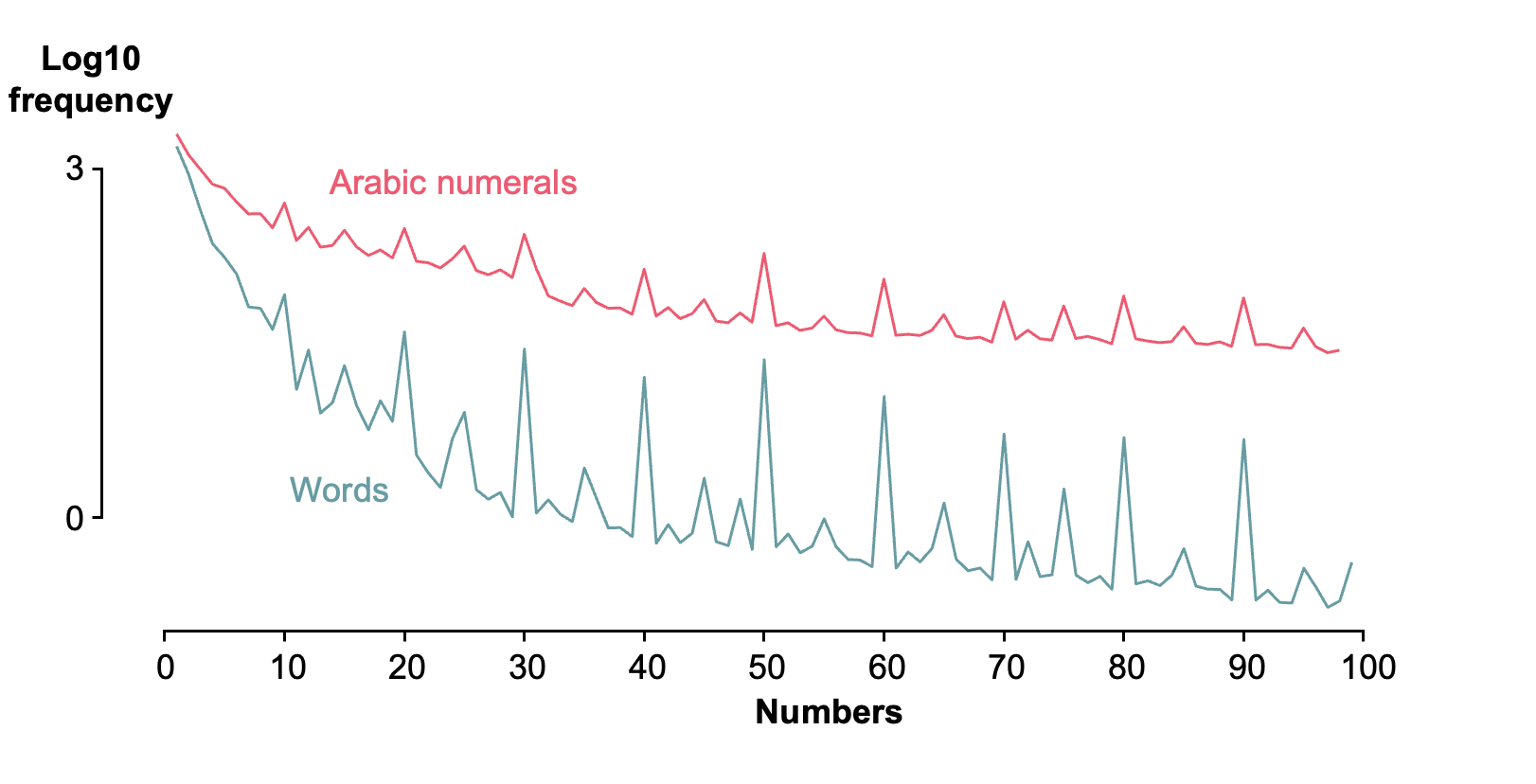


**Figure S2.** Comparison of the frequency of numbers presented either as words (as in the bulk of the paper) or as Arabic numerals, in English texts.


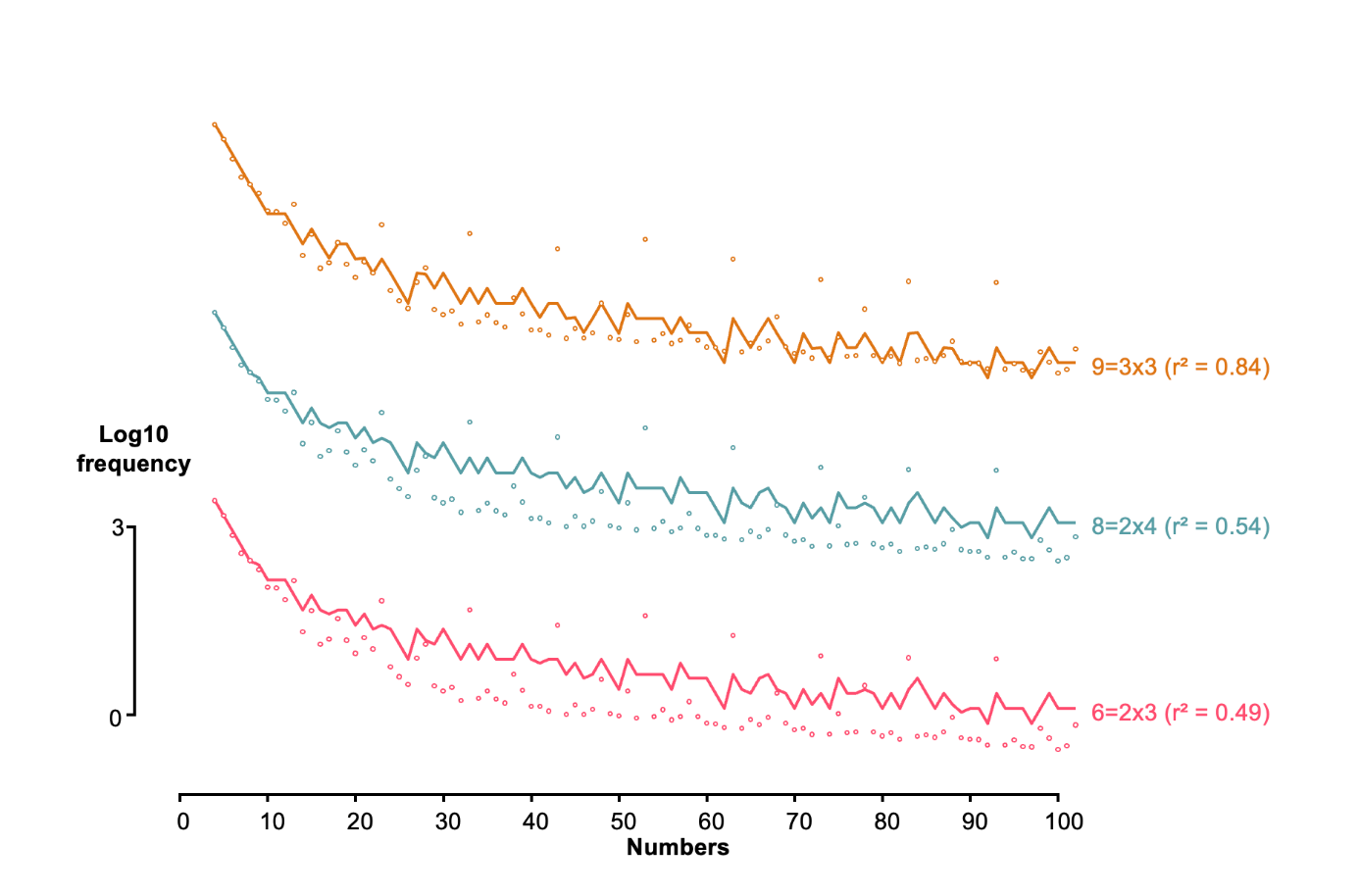


**Figure S3. Parameter-free fits of the shortest-path model to English number frequencies (red dots).** The entire number frequency curve could be approximately fit by setting the three free parameters of the model, using only the observed frequencies for four number words. Using the notation of figure 4, we set f1 as freq(1), cost_add_ = freq(2)/freq(1)^2^ (as derived from the expression 2=1+1), and c_mult_ as either freq(6)/(freq(3)×freq(2)), freq(8)/(freq(4)×freq(2)) or freq(9)/freq(3)², (as derived from the expressions shown on the right). In all case, the model nicely predict the overall trend and many of the local spikes of the observed frequency curve.


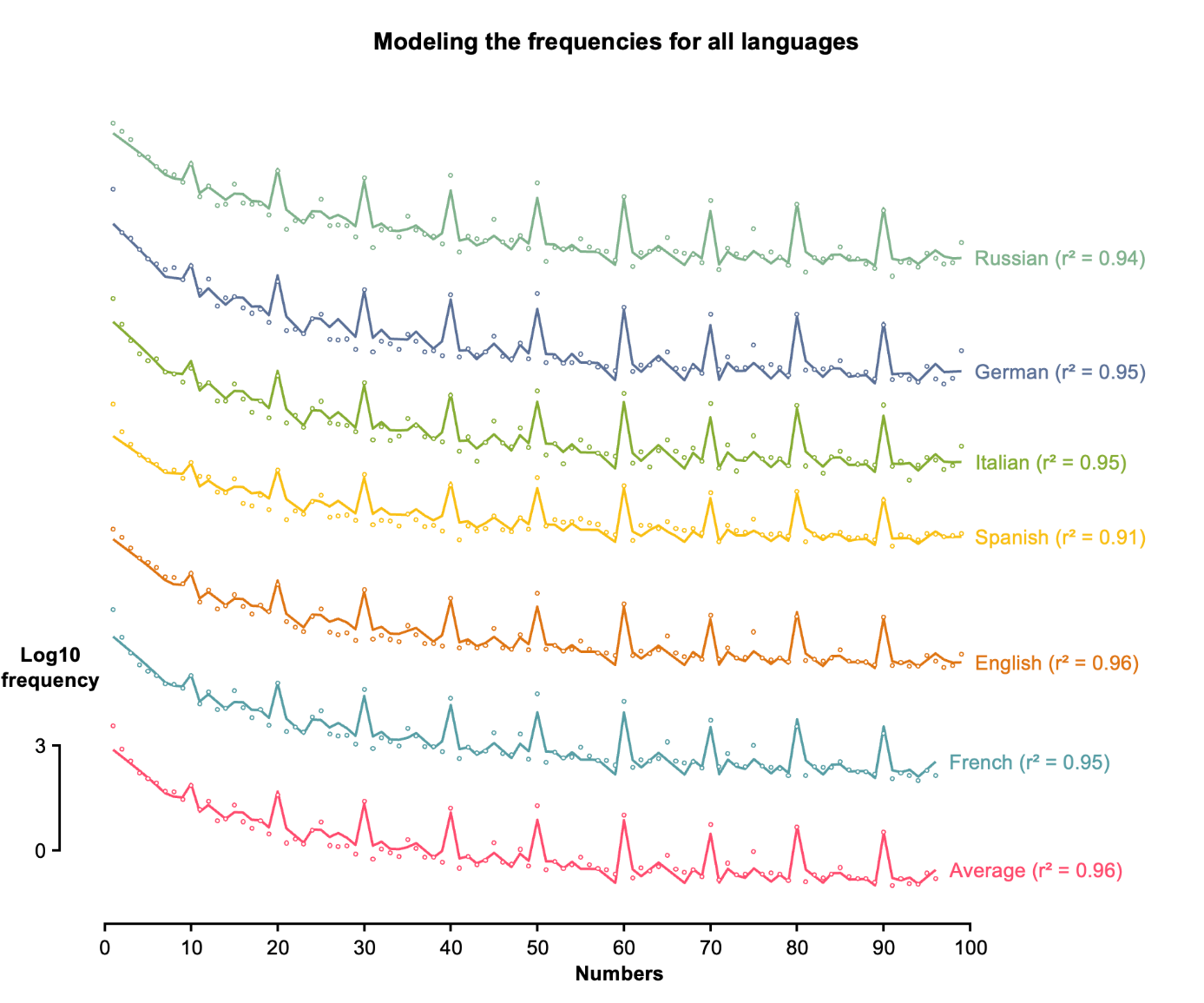


**Figure S4.** Predictions using the shortest path model with approximation to fit the log-frequencies in different languages.


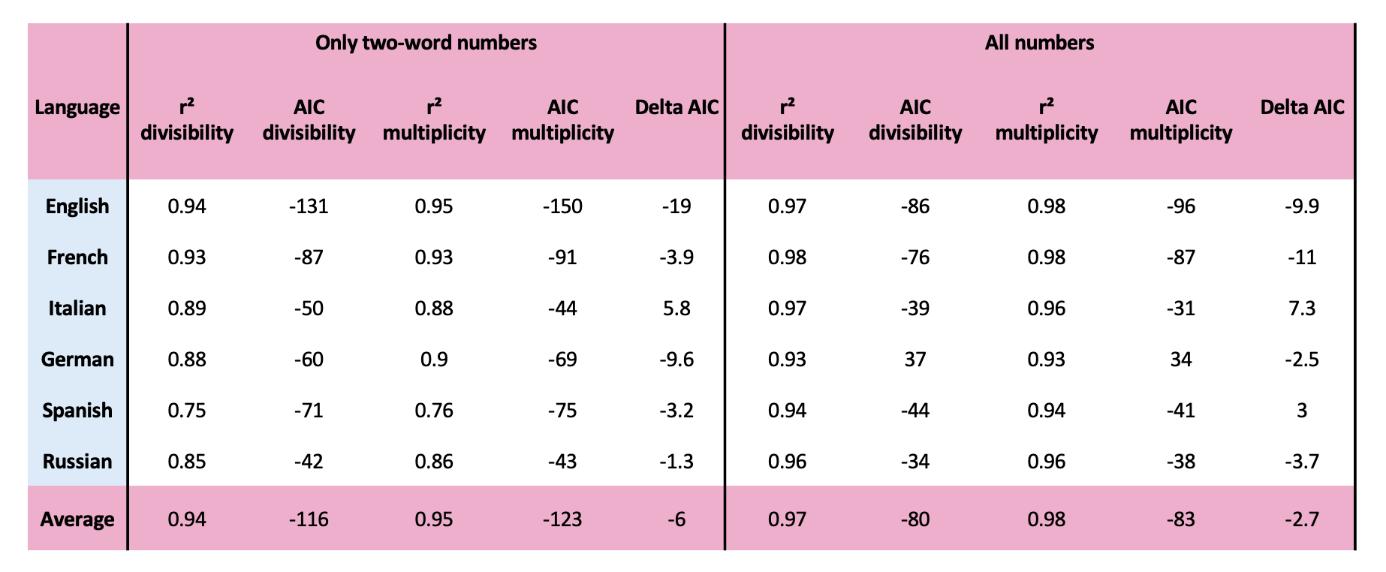


**Table S1.** Comparison of multiple regressions on Log10 frequency with Log10 number magnitude and either 4 indicator variables (0/1) for divisibility by 10, 5, 3 and 2, or 4 variables indicating the multiplicities of the same numbers 10, 5, 3, and 2 in the decomposition of the number into prime factors. The regression was either limited to 2-word numbers (left) or encompassed all numbers (right). In either case, the delta AIC criteria indicated that the multiplicity model did not outperform the divisibility model, except perhaps in English.
